# Comparing optical performance of a wide range of perovskite/silicon tandem architectures under real-world conditions

**DOI:** 10.1515/nanoph-2020-0643

**Published:** 2021-03-17

**Authors:** Manvika Singh, Rudi Santbergen, Indra Syifai, Arthur Weeber, Miro Zeman, Olindo Isabella

**Affiliations:** Delft University of Technology, PVMD group, Mekelweg 4, 2628 CD, Delft, The Netherlands; TNO Energy Transition, Solar Energy, Westerduinweg 3, 1755 LE, Petten, The Netherlands

**Keywords:** bi-facial, c-Si, optical modelling, perovskite, photocurrent, tandem

## Abstract

Since single junction c-Si solar cells are reaching their practical efficiency limit. Perovskite/c-Si tandem solar cells hold the promise of achieving greater than 30% efficiencies. In this regard, optical simulations can deliver guidelines for reducing the parasitic absorption losses and increasing the photocurrent density of the tandem solar cells. In this work, an optical study of 2, 3 and 4 terminal perovskite/c-Si tandem solar cells with c-Si solar bottom cells passivated by high thermal-budget poly-Si, poly-SiO_x_ and poly-SiC_x_ is performed to evaluate their optical performance with respect to the conventional tandem solar cells employing silicon heterojunction bottom cells. The parasitic absorption in these carrier selective passivating contacts has been quantified. It is shown that they enable greater than 20 mA/cm^2^ matched implied photocurrent density in un-encapsulated 2T tandem architecture along with being compatible with high temperature production processes. For studying the performance of such tandem devices in real-world irradiance conditions and for different locations of the world, the effect of solar spectrum and angle of incidence on their optical performance is studied. Passing from mono-facial to bi-facial tandem solar cells, the photocurrent density in the bottom cell can be increased, requiring again optical optimization. Here, we analyse the effect of albedo, perovskite thickness and band gap as well as geographical location on the optical performance of these bi-facial perovskite/c-Si tandem solar cells. Our optical study shows that bi-facial 2T tandems, that also convert light incident from the rear, require radically thicker perovskite layers to match the additional current from the c-Si bottom cell. For typical perovskite bandgap and albedo values, even doubling the perovskite thickness is not sufficient. In this respect, lower bandgap perovskites are very interesting for application not only in bi-facial 2T tandems but also in related 3T and 4T tandems.

## 1 Introduction

Crystalline silicon (c-Si) solar cells dominate the photovoltaic market due to their relatively high efficiency, low manufacturing costs and long-term stability. However, single-junction c-Si solar cells are reaching their practical efficiency limit of about 27% [1], [2]. One way to improve this efficiency limit is by stacking high bandgap cell on top of low bandgap cell in tandem configuration to reduce thermalization losses. Perovskite/perovskite [3], [4], [5], [6] and perovskite/c-Si [7], [8], [9], [10], [11], [12], [13], [14], [15], [16], [17], [18], [19], [20], [21], [22], [23], [24], [25], [26] tandem solar cells are gaining lot of attention in this regard. This is because perovskite solar cells have a sharp optical edge, a long diffusion length, a tuneable bandgap range and a good short wavelength response [27], [28], [29], [30]. These properties make perovskite-based solar cells ideal top cell in combination with c-Si bottom cell to form tandem solar cells. Several c-Si bottom cell technologies can be considered for this application. So far, most of the high efficiency tandem solar cells have been fabricated using silicon heterojunction (SHJ) bottom cell [7], [8], [9], [19], [20], [21], [22], [24], which are processed at temperatures well below 250 °C. On the other hand, widespread industrial c-Si solar cells are compatible with high temperature processes such as impurity gettering, thermal oxidation, dopants diffusion and firing through metallization. In this respect, perovskite/silicon-homojunction tandem cell has been demonstrated [31]. Carrier-selective passivating contacts (CSPCs) based on poly-Si [2], [32], [33], [34], [35], [36], [37], [38], poly-SiO_x_ [39], [40] or poly-SiC_x_ [41], [42] are typically formed at temperatures higher than 800 °C. Hence, they are excellent candidates for increasing the efficiency of high-thermal budget c-Si solar cells [35], [36], [43]. A potential drawback is their parasitic absorption, especially when they are deployed at the front side of the c-Si solar cell. These high temperature CSPCs have also been used in fabricating high-efficiency perovskite/c-Si tandem solar cells [14], [25].

In view of potential efficiencies well above 30%, perovskite/c-Si tandem solar cells with bottom cells passivated with different CSPCs can significantly reduce the levelized cost of electricity [44]. However, an important step in improving the efficiency of such tandem devices is by design optimization. Such design optimization can be performed by optical and electrical simulations. Researchers in the past have focussed on the optical simulations of perovskite/c-Si tandem solar cells with mostly silicon heterojunction c-Si solar cells [19], [45], [46], [47]. However, not much research is done regarding the optical simulations of perovskite/c-Si tandem solar cells with high temperature CSPCs. The above-mentioned issue of increased parasitic absorption in high-temperature CSPC occurs mostly in the shorter wavelength range (*λ* < 800 nm). It is therefore expected to be less severe in perovskite/silicon tandems where these shorter wavelengths are largely absorbed by the perovskite top cell before reaching the CSPCs. The parasitic absorption losses in high temperature CSPC in perovskite/silicon tandems have thus far not been quantified and this will be one of the objectives of this work. In this work, we have optically simulated perovskite/c-Si tandem solar cells with such high temperature CSPCs in GenPro4 software [48] and analysed different architectures, such as monolithically integrated two terminal (2T), three terminal (3T) and mechanically stacked four terminal (4T) perovskite/c-Si tandem solar cells. The performance of tandem solar cells endowed with poly-Si, poly-SiO_x_ or poly-SiC_x_ CSPCs are compared with that of tandem solar cells comprising heterojunction bottom solar cell. Finally, as these tandem solar cells are used in modules, where encapsulation materials such as glass and ethylene-vinyl acetate (EVA) are deployed, we have also studied the optical effect of encapsulation.

2T perovskite/c-Si tandem solar cells require current matching between the top perovskite solar cell and the bottom c-Si solar cell. The advantage of 4T tandem solar cell is that it does not require current matching. However, fabrication of top cell and bottom cell separately requires additional transparent contacts, which adds optical losses and fabrication costs. The pros and cons of mechanically stacked 4T tandem and monolithically integrated 2T are well documented [49], [50]. The advantages of both 2T and 4T configurations are combined in a 3T tandem configuration, which we consider as well. The 3T tandem configuration that we consider has one contact at the front and two contacts interdigitated at the rear [51]. Also, different tunnel recombination junction (TRJ) layers are studied to find the junction material with the most suitable optical properties for perovskite/c-Si tandem solar cells with CSPCs. Here, we have compared a wide range of tandem configurations and optimized the thickness of each layer to achieve maximum photocurrent current density and quantified the parasitic absorption losses in the high temperature CSPCs.

The optical simulations of perovskite/c-Si tandem solar cells are done at Standard Test Conditions (STC) [52]. However, in real-world operating conditions, the solar cell is not (always) illuminated perpendicularly, and the absorption of photons is influenced by the apparent position of the sun and spectral conditions [53], [54]. Here, we simulate and study the effect of different spectra and angles of incidence. In addition, we consider bi-facial tandem configurations, which can also convert the light incident on the rear side e.g. after ground reflection. For these solar cells, we study the effect of albedo, perovskite thickness and bandgap on the optical performance of the tandem solar cells for different locations in the world.

This contribution is organized as follows. We evaluate the potential of several 2T, 3T and 4T architectures and we study their optical behaviour in real-world conditions. Finally, we discuss the results and draw our conclusions.

## 2 Evaluation of optical potential of device architectures

**Figure 1:**
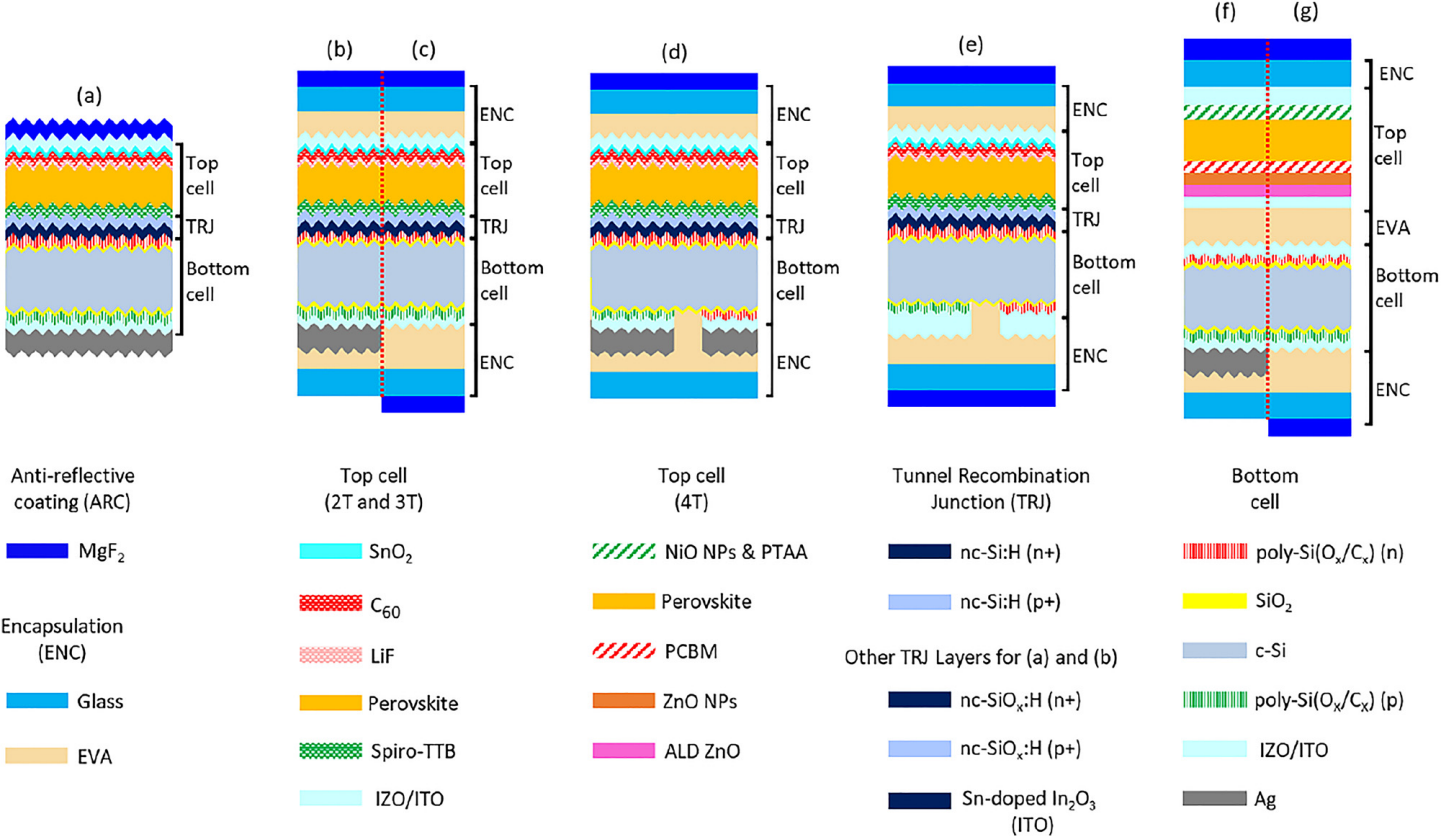
Simulated tandem structures. (a) 2T, (b) encapsulated 2T, (c) encapsulated 2T bi-facial, (d) encapsulated 3T, (e) encapsulated 3T bi-facial, (f) encapsulated 4T and (g) encapsulated 4T bi-facial.

Cell and module level modelling approaches and the modelling framework have been explained in the Supplementary Material. In this section, we use the validated optical model (see Supplementary Material) to quantify the implied photocurrent density of the perovskite/c-Si tandem solar cells. Our goal is to explain the subtle differences in implied photocurrent between (i) various electrical configurations (2T, 3T and 4T), (ii) various c-Si bottom cell architectures (with poly-Si, poly-SiO_x_ and poly-SiC_x_ CSPCs), and (iii) encapsulated and un-encapsulated tandems. An overview of the 2T, 3T and 4T perovskite/c-Si tandem structures simulated in GenPro4 are shown in Figure 1. Aside the case of the top cell in the 4T tandem configuration, for which it is still challenging to demonstrate a perovskite solar cell on a glassy textured substrate, all other configurations under test are endowed with textured surfaces. This choice was made not only to simulate the highest possible *J*_*ph*_ in tandem devices, but also to realize a flat broadband reflectance spectrum that allows for high optical performance also in encapsulated devices. Unless explicitly stated, simulated 2T and 3T tandem devices are endowed with a *p* ^+^-nc-Si:H/*n* ^+^-nc-Si:H stack that has the role of a TRJ, as in the validated 2T tandem solar cell reported in the study by Sahli et al. [8]. In this section, we consider “standard” illumination conditions (AM 1.5 spectrum and normal incidence). These results will lay the foundation for the analysis of real-world illumination conditions that will be considered in Section 3.

### 2.1 2T perovskite/c-Si tandem solar cells

**Figure 2:**
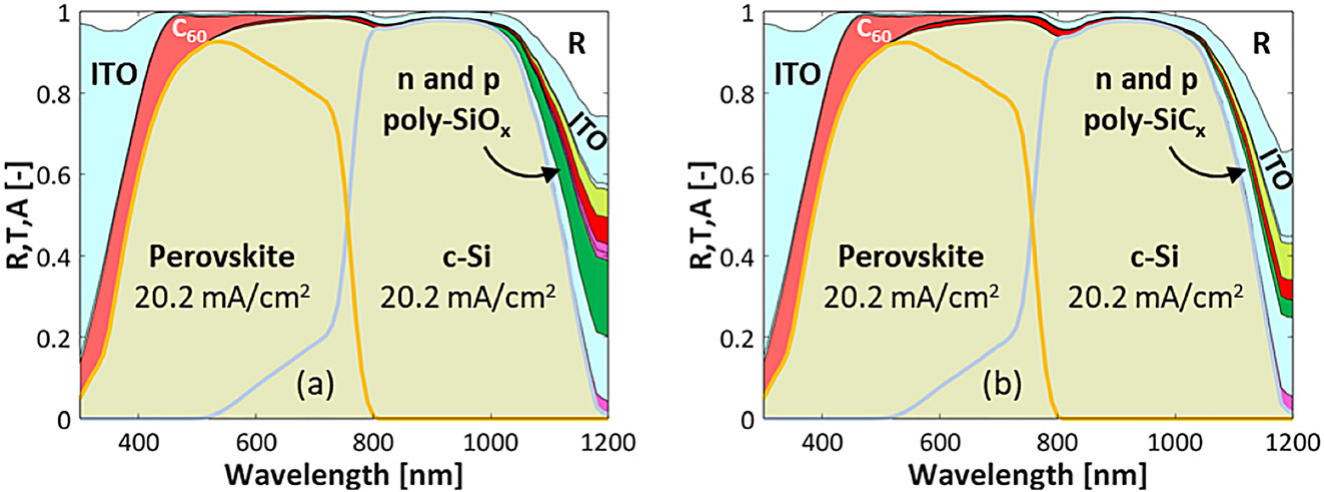
Reflectance and absorptance of 2T perovskite/c-Si tandem solar cell (see Figure 1(a)) with top cell as in the study by Sahli et al. [8] and bottom cell as (a) poly-SiO_x_ passivated c-Si solar cell or (b) poly-SiC_x_ passivated c-Si solar cell.

Referring to Figure 1(a), we compare here the optical performance of 2T tandem configurations comprising poly-Si, poly-SiO_x_ or poly-SiC_x_ CSPCs in the bottom cell and with simulation inputs, such as optical properties and thicknesses of each layer, as explained in Supplementary Material. Figure 2(a) shows the wavelength-dependent reflectance and absorptance spectra for the 2T tandem solar cell with poly-SiO_x_ passivated c-Si solar cell. The useful absorption in perovskite and c-Si solar cells is shown by orange and grey lines, respectively. Integrating these spectra over the AM 1.5 spectrum gives an implied photocurrent density of 20.2 mA/cm^2^, as the top and bottom cell photocurrent densities were perfectly matched by tuning the perovskite thickness to 545 nm. The white area represents the reflection loss, and the remaining coloured areas represent the parasitic absorption losses in supporting layers, such as transparent contacts (light blue) or electron transport layer (light red). The absorption losses in the *n*- and *p*-type poly-SiO_x_ CSPCs, indicated by the red and dark green areas, correspond to 0.32 and 0.63 mA/cm^2^, respectively. As anticipated, because the perovskite layer on top absorbs most shorter wavelength light (*λ* < 800 nm), these parasitic absorption losses in the poly-SiO_x_ CSPC are much lower than in case of a single junction device, especially for the light-facing n-type poly-SiO_x_ (see Supplementary Material).

When used in modules, these solar cells are encapsulated with glass and EVA (see Figure1(b)). The glass and EVA absorb 0.46 and 0.21 mA/cm^2^, respectively, and cause additional reflection losses from the air/glass interface. As a result, the implied photocurrent density after current matching is 19.49 mA/cm^2^, a reduction of 3.5% compared to the un-encapsulated case. Because the absorption in glass is slightly larger in the infrared compared to the visible part of the spectrum, a slightly lower perovskite thickness of 515 nm is now required to match the top and bottom cell currents.

A similar analysis was performed for 2T tandem solar cell with poly-SiC_x_ and poly-Si CSPCs. Figure 2(b) reports the wavelength-dependent reflectance and absorptance spectra for 2T tandem with poly-SiC_x_ passivated CSPCs. Again, the perovskite thickness is tuned to achieve a matched implied photocurrent density. The reference tandem with SHJ has the thinnest supporting layers (15-nm thick electron transport and hole transport stacks, see Supplementary Material), exhibiting the lowest parasitic absorption losses and thus giving the highest *J*_*ph*_. The poly-SiO_x_ is more absorbing than poly-SiC_x_ for wavelengths longer than 1000 nm, but slightly less absorbing for shorter wavelengths (see Figure 2(b)). These two effects compensate each other and thus numerically similar *J*_*ph*_ for poly-SiC_x_ and poly-SiO_x_ tandems is obtained. On the other hand, as both *n*-type and *p*-type poly-Si materials are less absorptive than their poly-SiO_x_ and poly-SiC_x_ counterparts, the performance of the tandem with poly-Si CSPCs is higher than that of the other high-thermal budget CSPCs but is still slightly lower than the SHJ reference. We fixed a priori the thickness of the high-thermal budget CSPCs to comply with the typical architecture of reported devices in literature that do not show fill factor issues. Given the full planar deployment of such layers in tandem devices and their contact with the TRJ on one side and with the transparent conductive oxide (TCO) on the other side, it could be optically beneficial to attempt their thinning. However, we leave this sensitivity study to a more experimental setting for also verifying the electrical behaviour of the resulting devices. Table 1 gives an overview of the perovskite thickness with the matched implied photocurrent density achieved in the 2T tandem with different CSPCs in both un-encapsulated and encapsulated cases.

**Table 1:** Implied photocurrent density (*J*_*ph*_) of 2T tandem with different CSPCs.

TRJ	Un-encapsulated		Encapsulated	
	*J*_*ph*_ matched (mA/cm^2^)	Perovskite thickness (nm)	*J*_*ph*_ matched (mA/cm^2^)	Perovskite thickness (nm)
	*p* ^+^-nc-Si:H/*n* ^+^-nc-Si:H			

SHJ (ref.)	20.41	560	19.70	535
Poly-Si	20.34	560	19.64	527
Poly-SiO_x_	20.20	545	19.49	515
Poly-SiC_x_	20.20	535	19.53	510

	***p* ^+^-nc-SiO_x_:H/*n* ^+^-nc-SiO_x_:H**			

SHJ	20.41	550	19.70	520
Poly-Si	20.34	550	19.65	523
Poly-SiO_x_	20.20	533	19.50	505
Poly-SiC_x_	20.20	520	19.53	500

	**Sn-doped In_2_O_3_ (ITO)**			

SHJ	20.36	540	19.66	515
Poly-Si	20.28	540	19.61	515
Poly-SiO_x_	20.14	520	19.45	497
Poly-SiC_x_	20.15	510	19.50	490

	**No tunnel recombination junction**			

SHJ	20.44	568	19.72	537
Poly-Si	20.39	565	19.66	535
Poly-SiO_x_	20.22	547	19.51	517
Poly-SiC_x_	20.24	541	19.56	514

SHJ stands for silicon heterojunction and it is intended as reference.

#### 2.1.1 Tunnel recombination junction

The tunnel recombination junction is an important part of the 2T tandem solar cell. From the optical point of view, the tunnel recombination layers should be transparent to the light transmitted by the perovskite absorber so that it can reach the bottom c-Si solar cell. Also, the real part of refractive index should be in between the refractive indices of the layer above and below of the TRJ (in this case Spiro-TTB and n-type doped poly-Si, poly-SiO_x_ or poly-SiC_x_). The electrical requirements of tunnel recombination junction are important as well [55], but they are outside the scope of this optical study. Results so far rely on doped nc-Si:H layers [8] used as TRJ. In this section, we explore the possibility of using nc-SiO_x_:H [56], [57], [58] or Sn-doped In_2_O_3_ (ITO) TCO as TRJ layers (see Table 1). We find that by using nc-SiO_x_:H as TRJ, *J*_*ph*_ values nearly the same as those computed for tandems with nc-Si:H TRJ are obtained. This is due to two factors cancelling out: (i) the nc-SiO_x_:H absorbs less compared to nc-Si:H, while (ii) the nc-SiO_x_:H has a refractive index value less ideally situated between Spiro and CSPC, which increases the reflection losses slightly. Note that because of this increased reflectance, perovskite layers have to be used that are 10–15 nm thinner. The TCO-based TRJ, on the other hand, parasitically absorbs more than the other two TRJs, so the matched *J*_*ph*_ is lower and this trend is observed for all the four CSPCs considered in Table 1. It also includes the case of direct contact between spiro-TTB (hole-transporting material of the perovskite) and CSPCs. We observe that in this case, a slightly higher matched implied photocurrent density can be achieved due to less parasitic absorption in TRJ. However, the electrical performance of such tandems without TRJ would need further electrical investigations.

### 2.2 3T perovskite/c-Si tandem solar cell

As shown in Figure 1(d), the 3T tandem solar cell is optically very similar to the 2T tandem, but it has a c-Si IBC solar cell as the bottom cell. Thus, at the rear side of the device, there are alternating *n*-contact, gap, *p*-contact, gap, etc. These parts have slightly different optical properties. Since GenPro4 is a 1D simulator, separate simulations are performed for each of the *n*-contact, *p*-contact and gap regions. The calculated absorptances are then combined into a weighted average, where the relative surface areas of *n*-contact (20%), *p*-contact (70%) and gap (10%) are used as the respective weights. Just as for the 2T tandem, the contacts of the 3T tandem can be endowed with poly-Si, poly-SiO_x_ or poly-SiC_x_.

In 3T configuration no current matching between top and bottom cell is required. In other words, increasing the thickness of the perovskite absorber layer, the implied photocurrent density of the overall device could be increased. In our simulation campaign, however, we kept the thickness of the perovskite absorber layer fixed at the value found to get current matching in 2T configuration (see Table 1). This choice was (i) to prevent the proposal of 3T devices with an overly thick top absorber, which could result in experimental issues, and (ii) to compare the performance of 3T tandems with their 2T configuration counterparts (see Section 4.1). Running our optical model, we found that the influence of TRJ layers for all cases of c-Si bottom cells on the optical performance of 3T tandem devices was negligible and due to the decoupling of *J*_*ph*_ between top and bottom cells. In this contribution we then report only the results related to the *p* ^+^-nc-Si:H/*n* ^+^-nc-Si:H TRJ as in the study by Sahli et al. [8].

**Figure 3:**
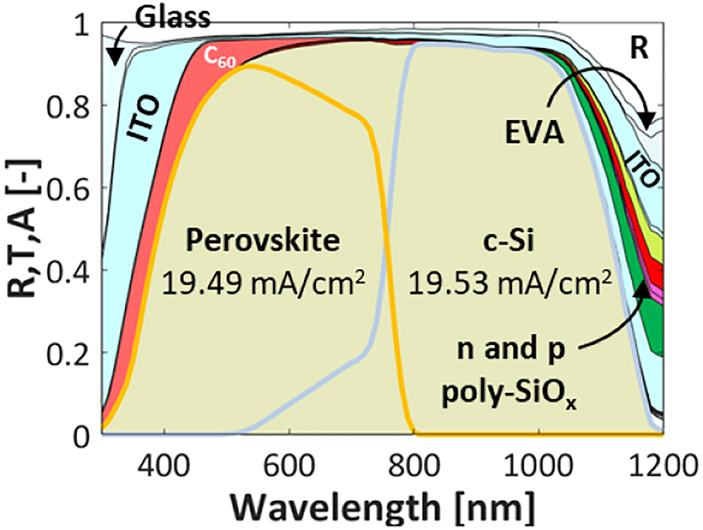
Reflectance and absorptance of 3T encapsulated perovskite/c-Si tandem solar cell (see Figure 1(d)) with top cell and TRJ as in the study by Sahli et al. [8] and a c-Si IBC bottom cell passivated with poly-SiO_x_ CSPCs.

As an example, the wavelength dependent reflectance and absorptance spectra for an encapsulated 3T tandem with poly-SiO_x_ passivated c-Si bottom solar cell are given in Figure 3. Aside the broadband increase in reflectance due to the presence of glass, we observe that this 3T tandem device exhibits an optical behaviour very similar to the un-encapsulated 2T tandem device reported in Figure 2(a). Deploying an IBC architecture for the c-Si bottom cell, for which both contacts based on poly-SiO_x_ are placed at the rear of the tandem, one could expect a lower absorption loss in such layers with respect to the 2T tandem counterpart. However, this is not case because the top perovskite cell behaves optically very similarly as in the 2T tandem, absorbing most of the light before 800 nm and still exposing at longer wavelengths the parasitically absorptive behaviour of the poly-SiO_x_ layers. Very similar results are obtained in case of poly-Si and poly-SiC_x_ technologies. An overview of all the results with different CSPCs and for different tandem configurations are given in Table 2.

**Table 2:** Implied photocurrent density of encapsulated mono-facial tandem devices with c-Si solar cells passivated with poly-Si, or poly-SiO_x_ or poly-SiC_x_.

	*J*_*ph*_ [mA/cm^2^]								
	*d*_pero_ [nm]	2T	3T		4T Textured top cell		*d*_pero_ [nm]	4T Flat top cell	
			Top	Bottom	Top	Bottom		Top	Bottom
SHJ (ref.)	535	19.70	19.72	19.63	19.60	19.02	513	19.38	18.58
Poly-Si	527	19.64	19.62	19.62	19.52	18.99	513	19.36	18.53
Poly-SiO_x_	515	19.49	19.49	19.53	19.56	18.84	513	19.35	18.25
Poly-SiC_x_	510	19.53	19.53	19.51	19.54	18.84	513	19.38	18.23

Tandem devices endowed with SHJ bottom cell are reported as reference. The 4T architecture with textured top cell refers to Figure 1(f) but with textured top cell as in Figure 1(b); *d*_pero_ stands for thickness of the perovskite absorber and in case of 2T it is the thickness for which current matching is achieved.

### 2.3 4T perovskite/c-Si tandem solar cell

In the 4T perovskite/c-Si tandem solar cell, the top and bottom cells are mechanically stacked; thus, they are connected optically but not electrically. Similar to the previous case of 3T tandem configuration, the thickness of the perovskite absorber layer is fixed at 513 nm, which is the value found when validating our modelling platform with the device reported in the study by Zhang et al. [59] (see Figure 1(f)).

**Figure 4:**
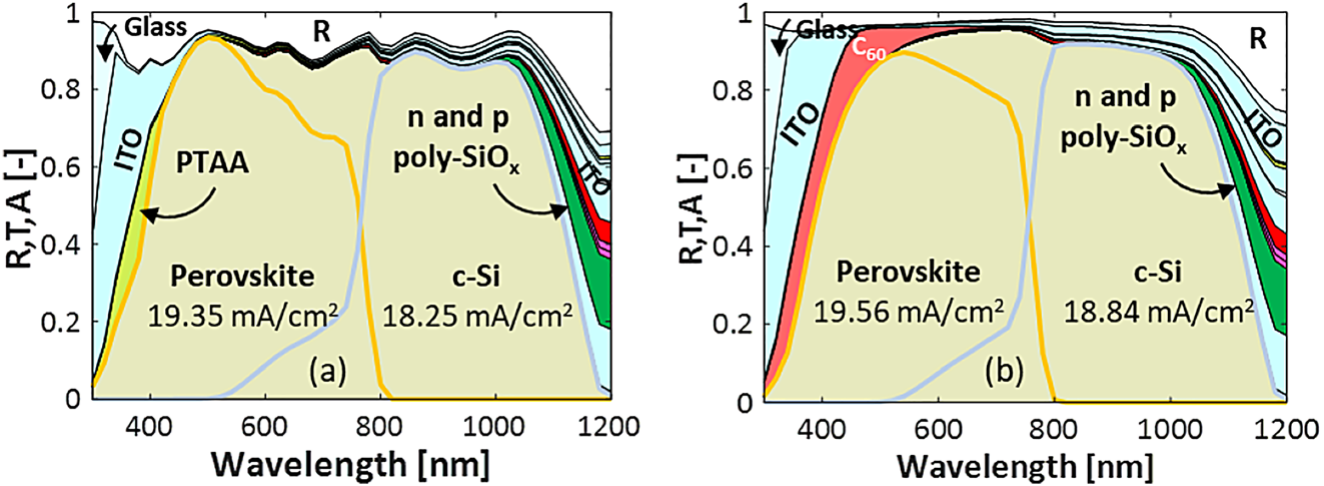
Reflectance and absorptance of 4T perovskite/c-Si tandem solar cell with (a) flat top cell as in the study by Zhang et al. [59] (see Figure 1(f)) or (b) textured top cell as in the study by Sahli et al. [8] (Figure 1(f) but with textured top cell as in Figure 1(b)). The c-Si bottom cell is passivated with poly-SiO_x_ CSPCs.

From an optical point of view, the main difference with 2T and 3T configuration is that in the 4T configuration the perovskite top cell is deposited front-to-back on a *flat* glass substrate while for the 2T and 3T configurations the perovskite top cell is deposited back-to-front on a *textured* c-Si bottom cell. From a fabrication point of view, it is so far challenging to fabricate a textured perovskite cell on glass. Figure 4(a) shows the reflectance and absorptance spectra for an encapsulated 4T tandem with poly-SiO_x_ passivated c-Si bottom solar cell. This diagram reveals that the flat top cell gives rise to significantly higher reflection losses (white area). Also, next to the *n*-type and *p*-type poly-SiO_x_ layers, which absorb around 0.29 and 0.61 mA/cm^2^, respectively, the additional ITO layer at the rear side of the top cell results in an increase of parasitic absorption losses. The *J*_*ph*_ of top and bottom cells are therefore reduced to 19.35 and 18.25 mA/cm^2^, respectively. In case of other CSPCs, a slight increase in bottom cell currents is observed due to lower free carrier absorption in the longer wavelengths (see Table 2).

Although practically difficult to manufacture, into an attempt to increase the *J*_*ph*_ in the top cell, we imagined texturing the top perovskite solar cell. For comparing the 2T, 3T and 4T configurations, we considered the textured perovskite cell from the study by Sahli et al. [8] (see Figure 4(b)). Owing to the texturing, the *J*_*ph*_ of the top cell increases, but the *J*_*ph*_ of the bottom cell is 18.84 mA/cm^2^, which is lower than that of 2T tandem due to additional losses in ITO layers. Table 2 gives an overview of the *J*_*ph*_ of the various encapsulated tandem configurations for different CSPCS.

## 3 Real-world conditions

The results presented thus far assume normal incidence and AM 1.5 spectrum for the incident light. However, in real-world conditions both the angle of incidence and spectral conditions vary simultaneously as the apparent sun position and cloud coverage vary with the time of day and year. In this section, we first study the individual effects of air mass (AM) and the angle of incidence (AOI) on the implied photocurrent density. To illustrate these effects, we consider the encapsulated 2T perovskite/c-Si tandem with bottom cell passivated with poly-SiO_x_ CSPC. We choose this CSPC because it is a new material whose optical performance in tandems has not been reported so far.

### 3.1 Air mass

**Figure 5:**
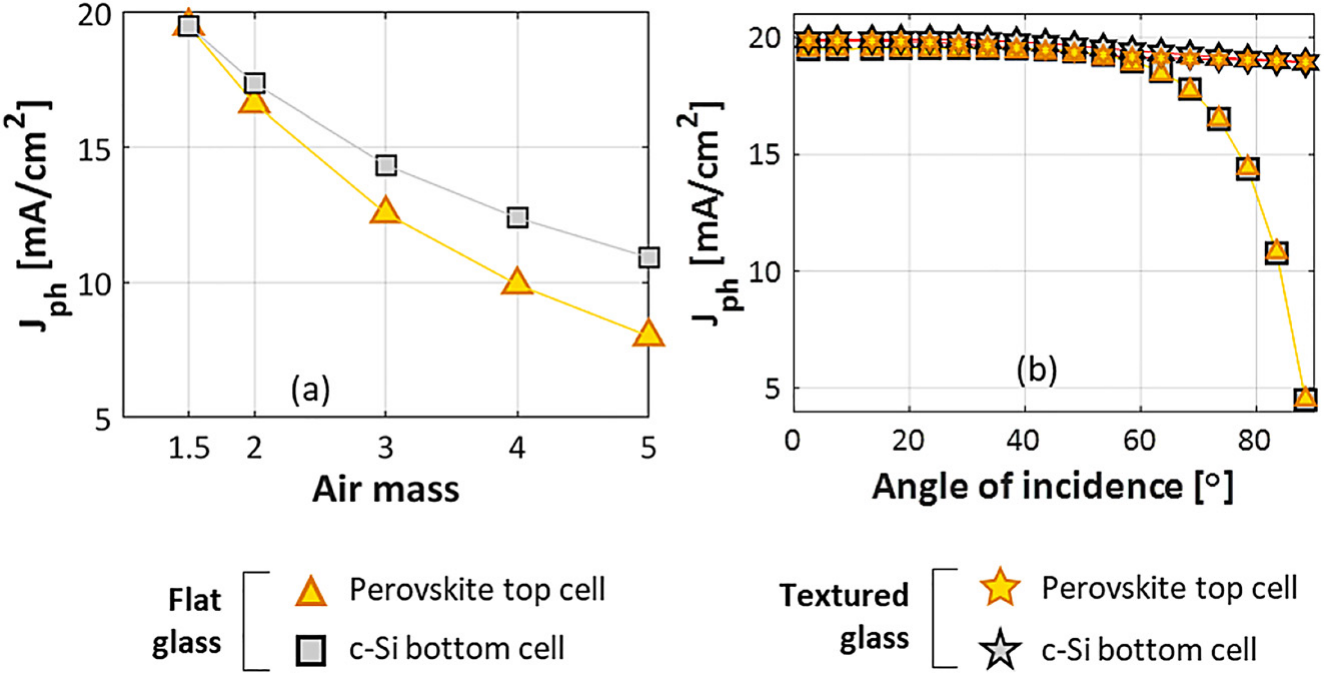
Implied photocurrent density of encapsulated 2T tandem with c-Si bottom cell passivated with poly-SiO_x_ as function of (a) AM and (b) angle of incidence.

When the sun’s elevation angle above the horizon decreases, the sunlight travels longer distance through the atmosphere, resulting in an increasing AM and corresponding reduction of the spectral irradiance, especially for the shorter wavelengths [52]. The spectra for different air mass are generated using the Simple Model of the Atmospheric Radiative Transfer of Sunshine (SMARTS) v2.9.2 developed by NREL [60]. In Figure 5(a), the effect of AM on the *J*_*ph*_ is shown for the encapsulated 2T poly-SiO_x_ passivated tandem solar cell. Here, we kept the perovskite thickness fixed at the thickness that matches the *J*_*ph*_ for the AM 1.5 spectrum. The graph shows that with increasing the AM, the implied photocurrent density in both perovskite and c-Si decrease. This is expected as the higher AM spectra have lower irradiance. The implied photocurrents of perovskite top cell and c-Si bottom cell do not decrease at the same rate, causing a current mismatch up to 35% between the top and the bottom cell as the spectrum varies. This effect is especially relevant for 2T tandems, but not for 3T and 4T tandems, which do not require current matching. Note that very similar behaviour was found also in case of tandems with c-Si bottom cells passivated with the other CSPCs considered in this work.

### 3.2 Angle of incidence

Unless the PV module is tracking the sun, the angle of incidence, between the incoming light and the PV module surface normal, also varies with the time of day and year. For increasing angle of incidence, one would expect higher reflection losses, especially at the air/glass interface. At the same time, the un-reflected light enters the solar cell under an oblique angle which might affect the optical path length in perovskite and silicon layers, potentially causing current mismatch. To study this effect on the implied photocurrent density, the angle of incidence is varied from 0° to 90°. The simulation result shown in Figure 5(b) reveals that with increasing the angle of incidence, the absorption in both perovskite and c-Si decreases thereby decreasing the corresponding implied photocurrent densities. Different than in case of the AM variation, the *J*_*ph*_ of top and bottom cell for flat glass encapsulation decrease at the same rate, remaining closely matched with changing angle of incidence. The maximum mismatch between the implied photocurrent densities is only 0.88%. Again, a very similar behaviour was found also in case of tandems with c-Si bottom cells passivated with the other CSPCs considered in this work. Light trapping effects due to the c-Si pyramid texture play an important role and are taken into consideration in our GenPro4 [48] model that is used to obtain the presented cell and module simulation results. In addition, textured glass simulations [61], [62] have been considered to study its effect on photocurrent density. Figure 5(b) reveals the behaviour of perovskite and c-Si solar cells in 2T configuration with textured glass encapsulation. The texture is on the outside of the glass. We observe that texturing increases the photocurrent density significantly especially at larger angles of incidence. The maximum mismatch between top and bottom cell’s implied photocurrent densities is below 1.5% in this case.

### 3.3 Effect of location on mono-facial tandem modules

Under real-world conditions the abovementioned variations in AM spectrum and angle of incidence occur simultaneously and vary depending on latitude and cloud coverage. We perform the hourly spectral irradiance analysis, as outlined in Supplementary Material, for the cities of Reykjavik (Iceland, 64°08′ N), Rome (Italy, 41°53′ N) and Alice Springs (Australia, 23°42′ S). We consider a module endowed with mono-facial 2T tandem solar cells and decorated with *p* ^+^-nc-Si:H/*n* ^+^-nc-Si:H TRJ.

To compare the year-averaged optical performance of different scenarios, we use the concept of the yearly average photocurrent density. For every hour of the year, we calculate the photon absorption rate in the individual perovskite and c-Si absorber layers. We assume that the tandem’s current output is limited by the sub-cell with the lowest implied photocurrent density, which for some hours will be the top cell while at other hours will be the bottom cell. We calculate this limiting implied photocurrent density for every hour of the year and then take its average. As this quantity is related to the annual energy yield, it allows us to optimize the optical design of the 2T tandem solar cell for a particular location without resorting to electrical yield calculations.

**Figure 6:**
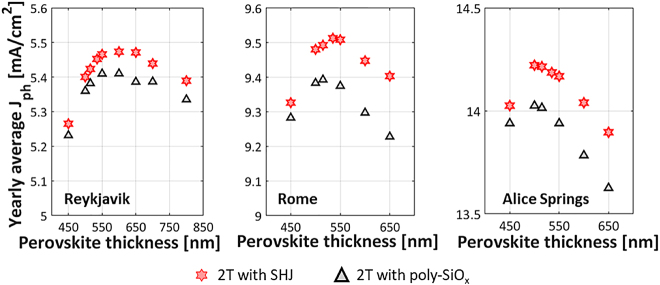
Yearly average photocurrent density of mono-facial 2T tandems with SHJ bottom cell (red stars) or c-Si bottom cell passivated with poly-SiO_x_ (grey triangles) for varying perovskite absorber thickness in (a) Reykjavik (Iceland), (b) Rome (Italy) and (c) Alice Springs (Australia).

Figure 6(a)–(c) shows the variation of the yearly average photocurrent of mono-facial 2T SHJ and poly-SiO_x_ passivated tandem solar modules with varying perovskite absorber layer thickness for the three above-mentioned locations. Under standard test conditions, the optimal perovskite thickness for these tandem solar cells are 535 and 515 nm, respectively. However, in real-world conditions for which the illumination varies over the year, we define the optimum thickness as the thickness that maximizes the yearly average photocurrent density We find that for different locations, the optimum thickness of the perovskite absorber changes considerably: ∼600 nm in case of Reykjavik for both c-Si technologies, 535 and 515 nm in case of Rome for SHJ and poly-SiO_x_ c-Si technologies, respectively, and 500 nm in case of Alice Springs for both c-Si technologies. We explain this by Rome having averaged over the year a similar spectrum as AM 1.5 and that is why the optimum thickness at STC is also the optimum for Rome. For Reykjavik, the spectrum has a higher AM, with relatively less visible light compared to infrared light, which must be compensated with a thicker perovskite. Alice Springs has a lower AM and so a thinner perovskite layer is the optimum. Optically, we observed that the tandem cell endowed with SHJ bottom c-Si cells outperforms that based on c-Si cells passivated with poly-SiO_x_ CSPCs (see Table 2). This result translates to module level (see Figure 6). Note that for perovskite thicknesses thinner than the optimum value, the perovskite current is for most hours of the year lower than that of c-Si and limits the tandem. Conversely, for perovskite thicknesses thicker than the optimum, the c-Si current limits the tandem.

**Figure 7:**
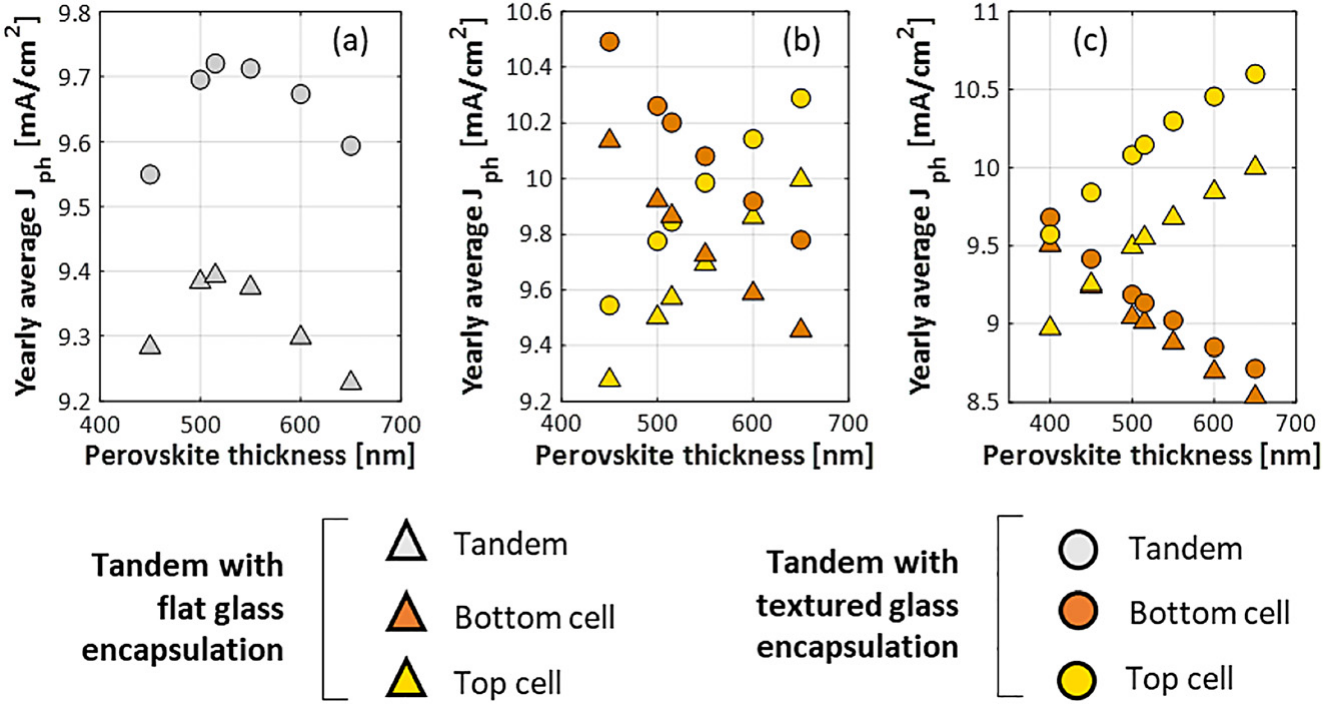
Simulated yearly average photocurrent density of mono-facial tandems with poly-SiO_x_ bottom cell for varying perovskite absorber thickness in Rome. The tandem configurations are (a) 2T, (b) 3T and (c) 4T.

In addition to 2T mono-facial tandem simulations, we also study 3T and 4T mono-facial tandem simulations, both for flat and textured glass encapsulation (as described in Section 3.2). The location considered here is only Rome. Figure 7(a)–(c) below shows the simulated yearly average photocurrent density of 2T, 3T and 4T mono-facial tandem solar cells. For 2T tandems we find that textured glass simulations give an increase in the yearly average photocurrent of about (3–4%) due to reduced reflection loss, especially for larger angles of incidence as shown in Figure 5(b). We also observe that on texturing the glass, the optimum thickness of the perovskite does not change. For 3T tandems, which have a similar structure, glass texture gives a similar (3–4%) enhancement for both top and bottom cell currents. In 4T tandems, the glass texture, besides reduced reflection loss, has a secondary effect of making light pass through the flat top cell more obliquely. This further enhances absorption in perovskite and increases the gain in top cell current to 6% compared to flat glass. The more oblique path does mean that a lower fraction of incoming photons is transmitted to the bottom cell, limiting the current gain in the bottom cell to 1%. Note that textured glass may increase the module soiling [63], a negative effect not taken into consideration in these simulations.

### 3.4 Effect of albedo and perovskite band gap on bi-facial tandem modules

**Figure 8:**
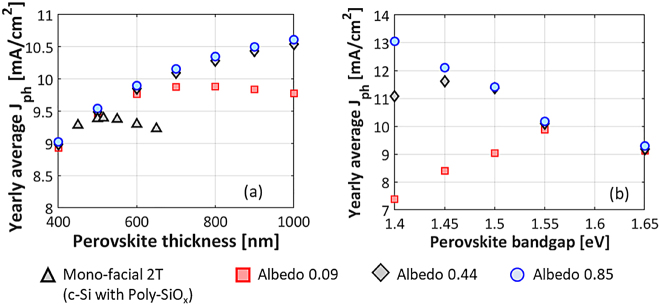
Yearly average photocurrent density of bi-facial 2T tandem module with c-Si bottom cell passivated with poly-SiO_x_ computed in Rome (Italy, tilt = 27°, azimuth = South) for different albedo values and as function of (a) perovskite absorber thickness or (b) perovskite absorber band gap (thickness fixed to 700 nm). The black triangles values are the reference from mono-facial 2T tandem cell; instead, red squares values are related to the mean albedo value of sandstone (0.09); the black diamonds to that of dry grass (0.44); and the blue circles to that of snow (0.85).

In bi-facial 2T tandem solar cells (see Figure 1(c)), the current in c-Si increases due to the reflection from the ground (albedo). Since the c-Si does not transmit any rear-incident light that can be absorbed by perovskite, the rear side irradiance does not contribute to additional current generation in the perovskite top cell. The effect of albedo on the optical performance of a bi-facial 2T tandem module with c-Si bottom cell passivated with poly-SiO_x_ has been studied. Mean albedo values of 0.09, 0.44 and 0.85 have been taken into consideration for different ground surfaces such as sandstone, dry grass and snow, respectively [64], [65]. Figure 8(a) below shows the yearly average photocurrent in the 2T bi-facial tandem for different albedo values as function of perovskite thickness. These values have been calculated for the location of Rome with a module tilt of 27° facing South and compared with the results for the mono-facial module (see grey symbols in Figure 8(a)). We observe that for low albedo values of 0.09 (= low impact of rear-incident light on the optical behaviour of the tandem), we get an optimum perovskite thickness between 700 and 800 nm. This is much thicker than the optimal 515-nm thick perovskite absorber found for the mono-facial tandem module; but it is still required to match the additional current density from the c-Si due to the rear side irradiance. Beyond 800-nm thick perovskite absorber, the c-Si current limits the tandem. On the other hand, for higher albedo values of 0.44 and 0.85, there is even more additional c-Si current due rear side irradiance, and the perovskite current seems to always limit the tandem, even in case of a 1000-nm thick perovskite layer.

To get current matching in case of mid-to-high level of albedo, another option is to reduce the optical bandgap of the perovskite absorber layer so that it can absorb a wider range of the solar spectrum. The optical bandgap of the perovskite has been blue-shifted in steps of 25 nm similar to the approach used in the study by Albrecht et al. [66]. To this end, we keep the location of Rome and the above-mentioned mounting configuration and we fixed to 700 nm the thickness of the perovskite absorber. We then determine the optimum bandgap corresponding to current matching. As reported in Figure 8(b), for bi-facial tandems with albedo value of 0.09, the optimum bandgap is (1.55 eV) whereas for 0.44 albedo, the optimum bandgap is 1.45 eV. For a very high albedo of 0.85 (snow), the optimum bandgap is less than 1.4 eV. As in Sections 3.1 and 3.2, a very similar behaviour was found also in case of tandems with c-Si bottom cells passivated with the other CSPCs considered in this work. However, we assume the ground to have a constant (wavelength independent) albedo. Therefore, the spectral distribution of light incident on the rear side is always the same as on the front side. In reality, the albedo of a material like grass is wavelength dependent [64], which will result in differences between front and rear spectra. This may introduce a minor deviation in calculated *J*_*ph*_ [67].

### 3.5 Effect of location on bi-facial tandem modules

**Figure 9:**
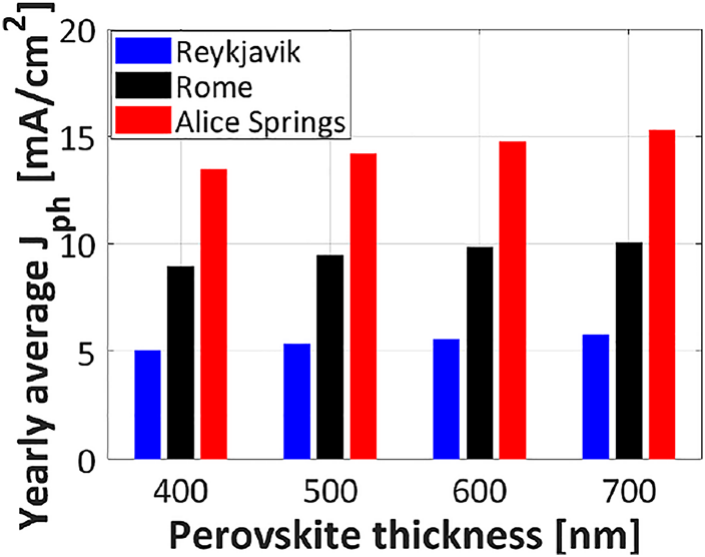
Yearly average photocurrent density of bi-facial 2T tandem with c-Si bottom cell passivated with poly-SiO_x_: comparison between different perovskite absorber thicknesses and different locations. Albedo value considered here is 0.44.

The analysis of perovskite thickness and bandgap in the previous section was presented for only one location and several albedo values. To thoroughly study the effect of location on the 2T bi-facial tandem modules, simulations have been performed also for Reykjavík in Iceland and Alice springs in Australia. Figure 9 shows a comparison of the yearly average photocurrent density for perovskite absorber thickness from 400 to 700 nm. Going from the least sunny to the sunniest location, the yearly average photocurrent density increases from Reykjavík to Rome to Alice Springs and for perovskite absorber layer thickness from 400 to 700 nm.

## 4 Discussion

### 4.1 Overall comparison between different perovskite/c-Si tandem architectures and CSPCs

The deployment of the poly-SiO_x_ CSPCs in single junction c-Si solar cells leads to significant additional parasitic absorption losses, which in turn reduces the implied photocurrent density (see Figure 3(a) of Supplementary Material). However, these optical losses occur largely for shorter wavelengths (*λ* < 600 nm) and do not play a critical role in perovskite/c-Si tandem configurations. In the 2T configuration, the implied photocurrent density reduction is limited to at most 0.3% for poly-Si, 0.8% for poly-SiC_x_ and 1% for poly-SiO_x_ CSPCs. The results for 2T, 3T and 4T tandems are summarized in Table 2. For 2T, the current matched top and bottom *J*_*ph*_ is given with the corresponding perovskite thickness. This shows that careful choice of the perovskite absorber thickness is crucial. For 3T and 4T, separate top and bottom *J*_*ph*_ are shown, considering for comparison the same perovskite thickness and texture as in 2T. The 3T and 4T tandem devices exhibit similar trends for the different CSPCs as 2T. Note that for the 3T tandem, the photocurrent density in the bottom cell can be tuned by varying the area of *n*-type and *p*-type doped layers at the rear as well as the gap in between them. In this respect, while the difference in absorption coefficient between *n*-type and *p*-type doped layers can be optically leveraged, one must take care that emitter-to-pitch ratio of the IBC device does not become too small, giving rise to charge collection issues [68]. For 4T, the bottom cell current is about 3% lower than that in 2T and 3T tandems due to parasitic absorption in additional ITO layers involved. When the perovskite top cell is not textured, the 4T configuration gives rise to additional reflection losses and correspondingly lower *J*_*ph*_ in both top and bottom cells.

Compared to tandem solar cells based on SHJ bottom cell, tandem solar cells based on poly-Si, poly-SiO_x_ and poly-SiC_x_ CSPCs have slightly lower photocurrent densities due to the higher parasitic absorption in these high temperature CSPCs. This is because they are thicker and more heavily doped than the low temperature counterparts. Despite this, they can still achieve greater than 20 mA/cm^2^ matched implied photocurrent density in the un-encapsulated architecture along with being compatible with high temperature production processes.

For 2T, it is needed to match the top and bottom *J*_*ph*_. For 3T and 4T, having a thicker perovskite absorber will lead to higher photocurrent in the top cell but lower photocurrent in the bottom cell. In these configurations, a larger perovskite thickness will give voltage output at a potentially higher current which will result in a higher efficiency. However, a very thick perovskite thickness such as around 1000 nm might lead to fabrication issues and to lower electrical properties such as *V*_*oc*_ because of a larger recombination triggered by limited diffusion length. For 2T tandem, the optimum perovskite thickness is crucial as by varying the perovskite thickness by 50 nm leads to a significant variation in photocurrent density (see Figure 7(a)). Figures 7(b) and (c) show the variation of photocurrent density with perovskite thickness for both top and bottom cell in 3T and 4T configurations. Since current matching is not required in 3T and 4T, even on changing perovskite thickness, the average photocurrent density is almost constant which comes around 9.7 mA/cm^2^ for 3T and 9.25 mA/cm^2^ for 4T (not shown).

### 4.2 Comparison of real-world conditions with standard test conditions

In the real-world conditions, the illumination spectrum and the Sun’s position change over the entire year. We observe that the optimum thickness of mono-facial 2T tandem with c-Si bottom cell passivated with poly-SiO_x_, under standard test conditions, is around 515 nm (see Table 2). This thickness is also optimum, averaged over the whole year, for the city of Rome. However, for a location with a somewhat higher AM spectrum such as Reykjavik, the optimum perovskite thickness is thicker (∼600 nm). The opposite is also true: for a location with a lower AM spectrum such as Alice Springs, the optimum perovskite thickness is thinner 500 nm (see Figure 6). Hence, the optimum thickness changes for the real-world conditions. So, in general, the spectrum and angle of incidence do influence the absorption in both mono-facial and bi-facial perovskite/c-Si tandem solar cell and related modules (see Figure 9). Like 2T tandem solar cells, also the 3T and the 4T tandem solar cells will show a varying yearly average photocurrent density with changing locations.

### 4.3 Comparison of mono-facial and bi-facial tandem solar cells

Compared to mono-facial tandems, a higher yearly averaged photocurrent density can be achieved with bi-facial tandems due to the ground reflectance, which contributes to the extra current in the bottom cell. For 2T tandems we observe that by increasing the thickness of perovskite absorber layer, the yearly averaged *J*_*ph*_ increases if current generation in perovskite is the limiting factor. This happens even for perovskite thicknesses as high as 1000 nm and for albedo values of 0.44 or 0.85 (see Figure 8(a)). The optimum thickness for 2T tandem is the point where the transition from perovskite to c-Si being the limiting factor in *J*_*ph*_ occurs. For a particular bandgap of perovskite absorber, its optimum thickness in bi-facial 2T tandems is higher than that in mono-facial tandems (see Figure 8(a)). Or conversely, for a particular perovskite thickness, the optimum bandgap in bi-facial 2T tandems is lower than that in mono-facial tandems (see Figure 8(b)). These effects are more pronounced the larger the albedo.

**Figure 10:**
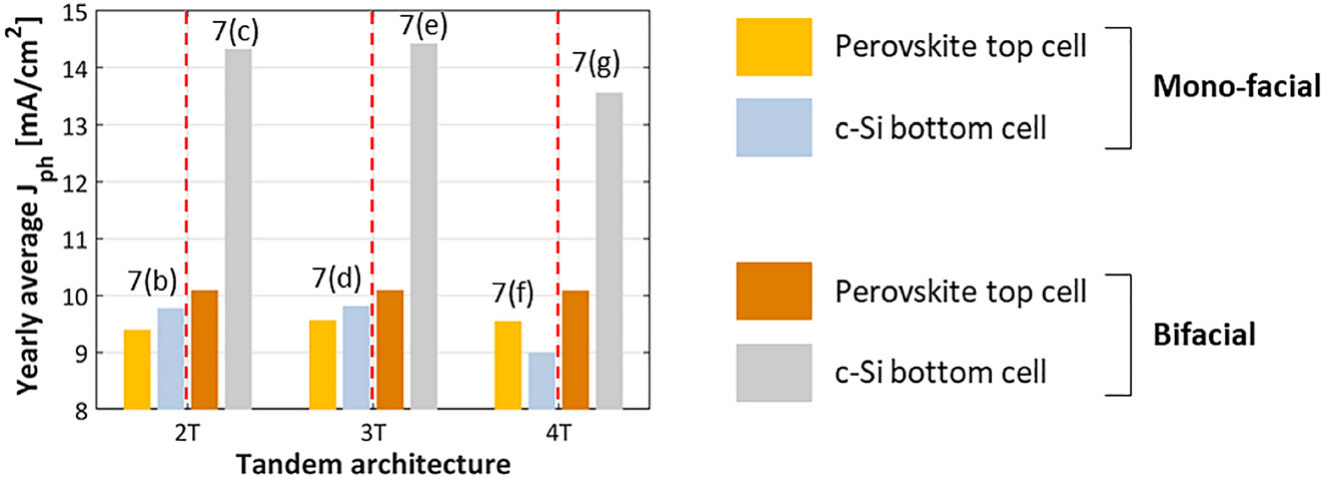
Comparison of yearly average photocurrent density of mono-facial and bi-facial 2T, 3T and 4T tandems with c-Si bottom cell passivated with poly-SiO_x_. Here bi-facial tandems are endowed with 700-nm thick perovskite absorber. Numbers and letters in the diagram refer to sub-figures in Figure 1.

To study the effect of tandem architectures, we compared the yearly averaged photocurrent density of mono-facial and bi-facial 2T, 3T and 4T tandem solar modules (see Figure 10). We choose the tandems based on poly-SiO_x_ passivated bottom cell as a comparative example. The mono-facial tandems were simulated for the optimized perovskite thickness of 515 nm in case of 2T and 3T architectures and 513 nm for 4T architecture (see Table 2). For bi-facial 2T tandems, since the perovskite current is the limiting factor for very thick absorber thickness, an upper value of 700 nm has been chosen (see Figure 8). On the other hand, 3T and 4T do not have the constraint of current matching. In these architectures, having a thicker perovskite absorber is beneficial because a higher current from the perovskite top cell will lead to higher efficiency. Hence, for bi-facial tandems, the perovskite thickness of 700 nm was chosen for comparison. To have a more realistic approach from a fabrication standpoint, flat top cell is chosen for the 4T given in Table 2.

From mono-facial to bi-facial tandems, the thicker perovskite absorber combined with the additional rear side absorption in the c-Si absorber, yield improved performance. Passing from 515-nm to 700-nm thick perovskite absorber (i.e. from mono-facial to bi-facial configurations), but still having perovskite as limiting factor, the yearly average photocurrent density of the top cell increases by 7.4, 5.5 and 5.5% in case of 2T, 3T and 4T tandem architectures, respectively. At the same time, the yearly average photocurrent density of the bottom cell increases by 46, 46 and 50% in case of 2T, 3T and 4T tandem architectures. This latter gain is due to the rear side absorption in c-Si, minus a small reduction in front-side absorption in c-Si due to the thicker perovskite layer.

For bi-facial 2T tandem, since the perovskite is limiting the total photocurrent density and thickness of perovskite cannot be increased further, lower bandgap perovskites should be considered to increase the average yearly photocurrent density. Note that changing the bandgap will also affect the electrical parameters of the tandem, such as the open circuit voltage, but this is out of scope for this optical study. For the 3T and 4T tandem configurations, the extra current in the bottom cell due to rear side absorption in c-Si contributes to the total photocurrent independent of the top cell current.

## 5 Conclusions

The goal of our work was to investigate what affects the optimum perovskite thickness for current matching in 2T perovskite/c-Si tandems and how the optical performance of these devices compares with that of related 3T and 4T tandems.

In 2T tandem configuration high temperature CSPCs such as poly-Si, poly-SiO_x_ and poly-SiC_x_ can achieve matched photocurrent density greater than 20 mA/cm^2^ and around 19.5 mA/cm^2^ without encapsulation and with encapsulation, respectively. These values are slightly lower than those of a 2T tandem based on SHJ bottom cell because of the high doping-driven free carrier absorption in high-thermal budget CSPCs. However, these CSPCs are compatible with high temperature production processes and are therefore appealing for the mainstream c-Si industry. We observed that when designing a 2T tandem, the effect of encapsulation should be taken into consideration as it reduces the optimum perovskite thickness by about 25 nm. With our modelling approach, we can also optimize the thickness of the perovskite absorber according to the location and angle of incidence, as the optimum thickness of the perovskite absorber under standard test conditions is not the same as in real-world conditions.

At module level, we introduced the yearly average photocurrent density as term of comparison among different tandem architectures. Evaluating bi-facial tandems, a higher yearly average photocurrent density than that of mono-facial tandems could be obtained due to the ground reflection that contributes extra current to bottom c-Si cell. The optimum thickness of the perovskite absorber for bi-facial tandems is higher than that of the mono-facial tandems. On the other hand, the bandgap of perovskite absorber is another parameter that can be tuned in bi-facial 2T, 3T and 4T tandems to harvest more current from the top cell without realizing an overly-thick top cells. For higher albedo values, the optimum bandgap of perovskite is lower as compared to the optimal bandgap found for lower albedo values. Careful optimization of albedo and thickness and bandgap of perovskite absorber is crucial in achieving a high yearly average photocurrent density.

## Supplementary Material

Supplementary Material
