# Supplementary material for: Comparing optical performance of a wide range of perovskite/silicon tandem architectures under real-world conditions
Source: Nanophotonics. 2021 Mar 17;10(8):2043–57. doi: 10.1515/nanoph-2020-0643 (PMC9646241; doi:10.1515/nanoph-2020-0643)
Supplement: Supplementary file 1 — Supplementary Material [file j_nanoph-2020-0643_suppl.docx]

Supplementary information for:

Comparing optical performance of a wide range of perovskite / silicon tandem architectures under real world conditions

M. Singh^1^, R. Santbergen^1^, I. Syifai^1^, A. Weeber^1,2^, M. Zeman^1^, O. Isabella^1^

1. Delft University of Technology, PVMD group, Mekelweg 4, 2628 CD Delft, the Netherlands

2. TNO Energy Transition, Solar Energy, Westerduinweg 3, 1755 LE Petten, the Netherlands

**1. Modelling approach**

**We first explain our cell-level and model-level modelling approaches and we validate our modelling framework.**

**1.1** **Cell level optical model**

Optical software GenPro4 [1], is used to perform optical simulations of perovskite/c-Si tandem solar cells. The generated output is reflectance (R), transmittance (T), absorptance (A) spectra of each layer of a solar cell and their related implied photocurrent densities (J_ph_). This is calculated by integrating the absorbed photons over the Air mass 1.5 (AM 1.5) [2]spectrum for standard test conditions.

The optical model combines wave and ray optics, as illustrated in Fig. 1(a) and 1(b) for the perovskite/ c-Si tandem solar cells. This means that it simultaneously takes into consideration the interference in the sub-wavelength layer stack of the perovskite top sub-cell, and the anti-reflective effects of the super-wavelength pyramid texture of the underlying c-Si bottom sub-cell.

GenPro4 is a one-dimensional (1D) simulator. It does not take into consideration the width of the device or optical shading by metal contact fingers. When the bottom cell is an interdigitated back contacted (IBC) solar cell, which has two interdigitated rear side contacts, the optical generation profile could vary across the width of the device. To simulate such cells, we have used a weighted averaging technique [3].

| 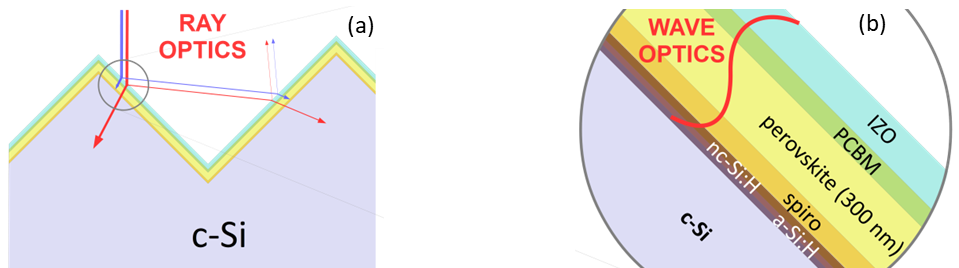 |
| --- |
| **Figure 1**: Working principles of GenPro4 optical model. (a) Ray optics is used to model the anti-reflective and light scattering effect of c-Si pyramid texture; (b) multi-layer wave optics is used to model the interference in the thin films deposited on top of the texture. |

**2.2** **Module level optical model**

Tandem cells in modules need to be encapsulated. This is reflected in our model by considering both glass and EVA materials. We assume that the simulated modules contain 10 rows of 6 cells. The width of a single cell is 15 cm, even though this dimension could be straightforwardly changed to accommodate larger silicon wafers dimensions. The mounting height is 0.5 m above the ground, tilting 27 degree and azimuth is South (North) for locations in Northern-hemisphere (Southern-hemisphere). Measured hourly global horizontal irradiance and direct normal irradiance data are input for the Perez sky model [4], which - combined with the hourly sun position - is used to recreate the real-world spectral irradiance conditions, considering both the direct and diffuse irradiance contributions. The ray-tracing method outlined in Ref. [5] is then used to calculate the plane of array irradiance. This method was extended for tandem solar cells. The method was made spectrally resolved and layer resolved, such that it can determine the individual photon absorption rate in all layers of the perovskite and silicon sub cells, considering the varying incident angles and illumination spectrum. For bifacial tandem solar cells, this calculation is performed separately for irradiance incident on front and for irradiance incident on the rear side of the PV module.

**3. Simulation input and validation**

The input of optical simulations in GenPro4 are complex refractive indices (*N* = *n* + i*k*), also known as *nk* data, and the thickness of each layer. For the bottom cell in 2T, 3T and 4T tandem, c-Si solar cells with poly-Si, poly-SiO_x_ and poly-SiC_x_ CSPCs have been considered. The refractive indices of poly-Si have been calculated for 1 $\times$20 cm^-3^ doping concentration [6]. For poly-SiO_x_ and poly-SiC_x_ carrier-selective passivating contacts, the refractive indices and extinction coefficients are measured using the inverse modelling (IM) technique [7]. The real and imaginary parts of these complex refractive indices are shown in Fig. 2(a) and (b). In the same diagrams, *nk* data of Ag and ITO are reported as in-house measured. The corresponding thickness are shown in table 1. To validate our simulations, we have compared the simulated absorptance with the external quantum efficiency (EQE) of front/back-contacted (FBC) c-Si solar cells with poly-SiO_x_ CSPCs (see Fig. 3). The simulated absorptance and the measured EQE are closely matched with an average deviation of less than 2%. These simulations have been performed in the wavelength range between 300 nm and 1200 nm. For single junction poly-SiO_x_ passivated c-Si solar cells, high parasitic absorption in the front n-doped poly-SiO_x_ layer is noticeable, which increases further when texturing is considered (not shown here).

| 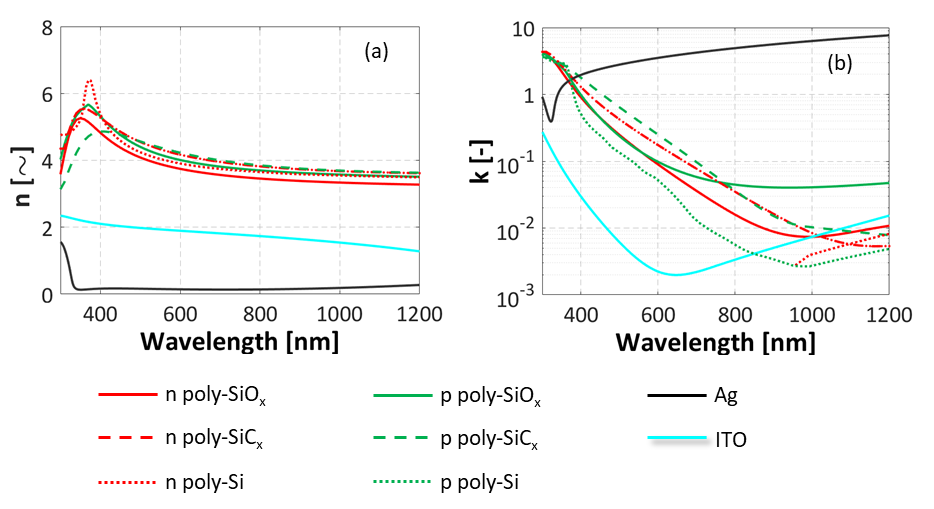 |
| --- |
| **Figure 2**: (a) Refractive index (n) and (b) extinction coefficient (k) of layers used in simulations (poly-Si, poly-SiO_x_ and poly-SiC_x_ CSPCs). |

The tandem structure given in Ref. [9] has been simulated and used as validation platform. The complex refractive indices used for simulations of perovskite/c-Si tandem solar cells are shown in Fig. 4, the corresponding thicknesses and the source of their *nk* data are shown in Table 1. The perovskite considered in our simulations is (Cs_0.05_(MA_0.17_FA_0.83_)_0.95_Pb(I_0.9_Br_0.1_)_3_) absorber layer from Ref. [10]. For the perovskite *nk* data that has been used in 2T simulations has been taken from Ref. [10], blue shifted by 20 nm to account for the slightly higher bandgap in Ref [9]. The reflectance of a bare air/glass interface is 4% and most of PV modules installed today employ some form of anti-reflective coating (ARC) to reduce this to less than 1%. In Ref. [9], MgF_2_ (magnesium fluoride) has been used as an ARC for 2T un-encapsulated tandem solar cell. To keep similar layer materials, we have used the same ARC (MgF_2_) on glass for all encapsulated tandem cases as well. However, MgF_2_ coatings are not sufficiently durable for real outdoor use [11]. Other materials such as porous silica [12] are typically used for outdoor applications. Since the typical refractive index of porous silica (1.3 – 1.4 depending on porosity) is in the same range as that of MgF_2_ (1.38), the optical effect of MgF_2_ coating that we use in our simulations is very similar to that of a porous silica coating (both reduce the air/glass reflectance to <1%). Ethylene Vinyl Acetate (EVA) has been used as an encapsulant in between the glass and the cell. The EVA used is EVAsky S87 from Brigdestone, a UV transmitting EVA. However, EVA does not have high enough barrier properties to protect perovskite against moisture, so real perovskite/silicon tandem modules would rather be encapsulated with polyolefins or ionomers. As these more novel encapsulation materials are still under development, we decided to use the optical constants of the abovementioned EVA instead.

| 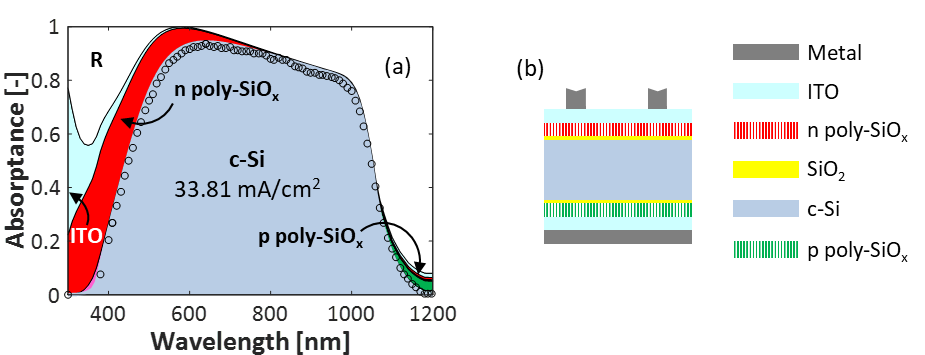 |
| --- |
| **Figure 3**: (a) Comparison of simulated absorptance in c-Si (grey area) with EQE (black circles) for flat/flat poly-SiO_x_ passivated c-Si solar cell (b) solar cell structure used for simulation and measurement of EQE [8]. |

For the textured surface, pyramids with a fixed base angle of 52 degrees and random position are given as input to the simulation. Fig. 5(a) shows that the simulated reflectance and absorptance spectra in perovskite and silicon absorbers are in excellent agreement with the measured reflectance, top cell EQE and bottom cell EQE, respectively, with average deviation of less than 1%. This shows that the GenPro4 model, combined with the input data, very accurately models this type of tandem device. The *nk* data and layer thicknesses of the perovskite top cell used in these validation simulations will be used to simulate the top cell optical behaviour in all subsequent 2T and 3T tandem simulations.

**Table 1**: Thickness of layers used in tandem simulations. Nps stands for nanoparticles.

| **2T,3T** | | **4T** | |
| --- | --- | --- | --- |
| **Top cell** | | **Top cell** | |
| **Layer** | **Thickness** | **Layer** | **Thickness** |
| MgF_2_ [13] | 134 | MgF_2_ [13] | 161 nm |
| IZO [14] | 110 nm | PTAA and NiO Nps [10] | 233 nm |
| SnO_2_ [3] | 10nm | ALD ZnO [10] | 28 nm |
| C_60_ [10] | 35 nm | PCBM [10] | 44 nm |
| Perovskite [2T,3T] [10] | *variable* | Perovskite [10] | 513 nm |
| Spiro-OMeTAD [15] | 12 nm | ITO (front and back) [10] | 142 nm and 237 nm |
| nc-Si (n and p) [measured in house] | 10 nm and 10 nm | ZnO Nps [10] | 17 nm |
| **Bottom cell** | | | |
| **Layer** | **Thickness** | ITO (front and back) [measured in house] | 75 nm and 120 nm |
| poly-Si(O_x_, C_x_) layers (n) [6],[7] | 30 nm | a-Si (n,p and i) [16] | 7,7 and 8 nm |
| c-Si bulk [14] | 280 μm | c-Si bulk [17] | 280 μm |
| poly-Si(O_x_, C_x_) layers (p) [6],[7] | 20 nm | Ag [measured in house] | 3 μm |
| **Encapsulation materials** | | | |
| Glass [18] | 3000 μm | EVA [19] | 300 μm |

| 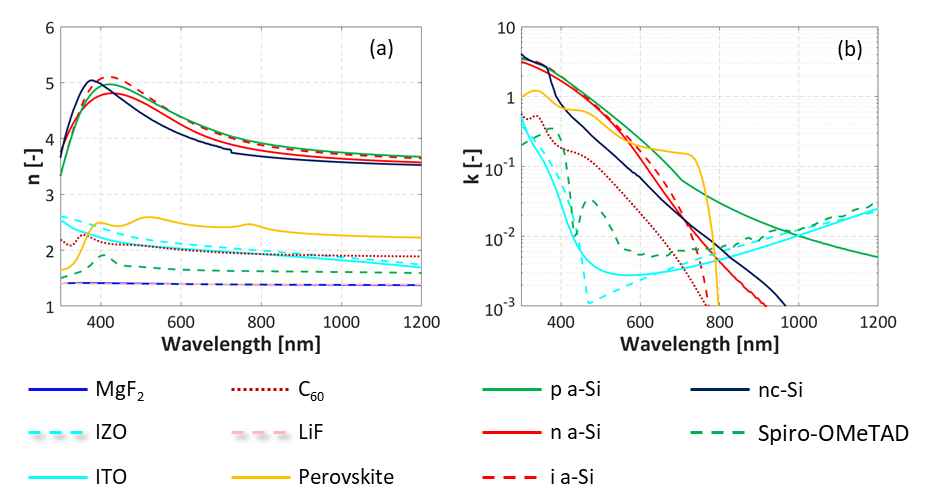 |
| --- |
| **Figure 4**: (a) Refractive index (n) and (b) extinction coefficient (k) of layers used to do simulations of 2T tandem given in Ref. [9]. The top cell data is used for all subsequent 2T and 3T simulations. |

| 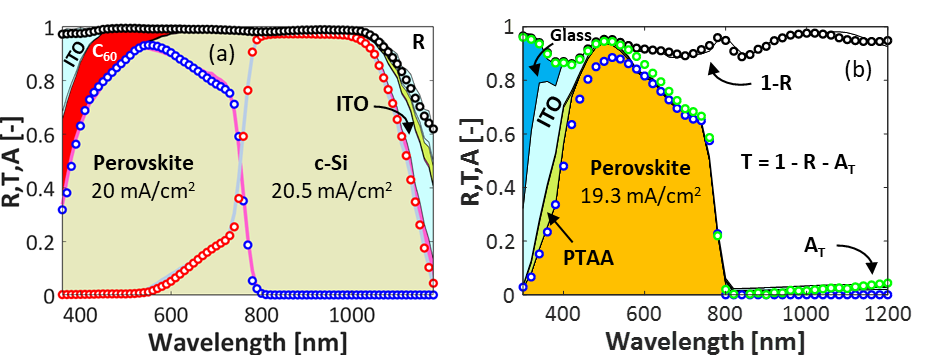 |
| --- |
| **Figure 5**: Comparison of measured EQE (top cell = blue circles, bottom cell = red circles), reflectance (R, black circles) and transmittance (T, between black circles and green circles) spectra with simulated R, T, and absorptance spectra in every layer (perovskite = pink line, c-Si = grey line): (a) 2T tandem from Ref. [9] and (b) transparent perovskite cell from Ref. [10]. A_T_ in (b) stands for total absorptance. |

For the 4T tandem simulations, the semi-transparent top cell structure has been taken from Ref. [10]. The complex refractive indices for the top cell are shown in Fig. 6. The thicknesses and the source of their *nk* data are shown in Table 1. The simulated reflectance, transmittance and absorptance spectra in perovskite absorber are shown in Fig. 5(b) and compared with the measured reflectance, transmittance and EQE spectra. Again, good agreement between simulation and measurement is obtained with an average deviation of only 1.3%. This shows that also for this architecture the optical model is validated; and the corresponding n and k data are accurate.

Textured glass simulations [20,21] have been considered to study its effect on photocurrent density. We considered 5 μm size pyramids textures both on the front and rear side of the glass. In reality, rear side texture should be of nano size [22] in order to allow deposition of good quality perovskite layer. However, simulating these rear side nano textures requires rigorous solving of Maxwell’s equations, not possible with our simulation approach [23].

| 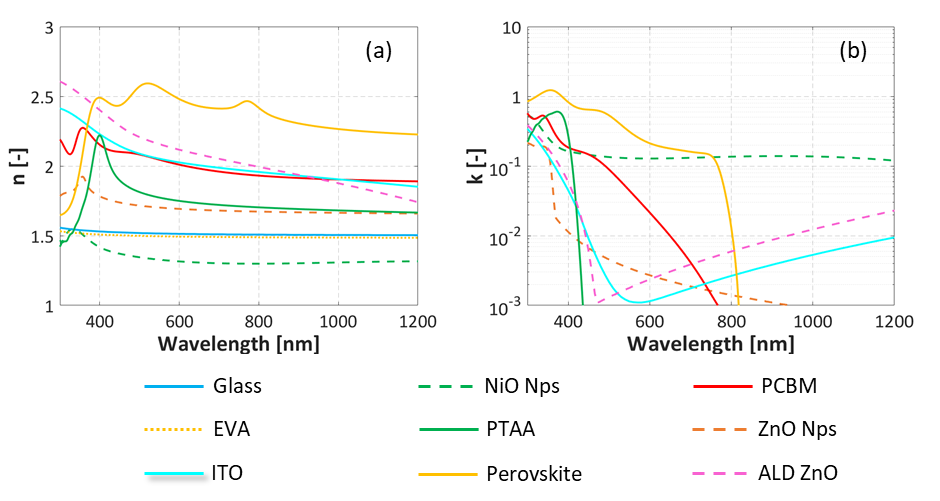  (a) |
| --- |
| **Figure 6**: (a) Refractive index (n) and (b) extinction coefficient (k) of layers used to do simulations of 4T tandem.  REFERENCES |

1. Santbergen R, Meguro T, Suezaki T, Koizumi G, Yamamoto K, Zeman M. GenPro4 Optical Model for Solar Cell Simulation and Its Application to Multijunction Solar Cells. IEEE J. Photovolt. 2017, 7, 919–926.
2. NREL Solar Spectra (Accessed December 3, 2020, at <https://www.nrel.gov/grid/solar-resource/spectra.html>)
3. Santbergen R, Uzu H, Yamamoto K, Zeman M. Optimization of Three-Terminal Perovskite/Silicon Tandem Solar Cells. IEEE J. Photovolt. 2019, 9, 446-451.
4. Perez R, Seals R, Michalsky J. All weather model for sky luminance distribution preliminary configuration and validation. Sol. Energy 1993, 50, 235-245.
5. Santbergen R, Muthukumar VA, Vackenborg RME, Van de wall WJA, Smets AHM, Zeman M. Calculation of irradiation distribution on PV modules by combining sky and sensitivity maps . Sol. Energy 2017, 150, 49-54.
6. Baker-Finch SC, McIntosh KR, Yan D, Fong KC, Kho TC. Near-infrared free carrier absorption in heavily doped silicon. J. Appl. Phys 2014, 116, 063106.
7. Singh M, Santbergen R, Mazzarella L et al. Optical characterization of poly-SiOx and poly-SiCx carrier-selective passivating contacts. Sol. Energy Mater. and Solar Cells 2020, 210, 110507.
8. Yang G, Guo P, Procel P, Weeber A, Isabella O, Zeman M. Poly-crystalline silicon-oxide films as carrier-selective passivating contacts for c-Si solar cells. Appl. Phys. Lett. 2018, 112, 193904.
9. Sahli F, Werner J, Kamino BA et al. Fully textured monolithic perovskite/silicon tandem solar cells with 25.2% power conversion efficiency. Nat. Mater. 2018, 17, 820-826.
10. Zhang D, Najafi M, Zardetto V et al. High efficiency 4-terminal perovskite/c-Si tandem cells. Sol. Energy Mater. Sol. Cells 2018, 188, 1-5.
11. Yan L, Liu N, Zhao S, et al. Effect of Hydrophobic Modification on the Durability and Environmental Properties of Porous MgF2 Antireflective Films. Acta Metallurgica Sinica 2014, 27, 649-655.
12. Agustín-Sáenz C, Machado M, Nohava J et al. Mechanical properties and field performance of hydrophobic antireflective sol-gel coatings on the cover glass of photovoltaic modules Sol. Energy Mater. Sol. Cells 2020, 216, 110694.
13. Dodge MJ. Refractive properties of magnesium fluoride. Appl. Optics 1984, 23, 1980-1985.
14. Werner J, Dubuis G, Walter A et al. Sputtered rear electrode with broadband transparency for perovskite solar cells Sol. Energy Mater. Sol. Cells 2015, 141, 407-413.
15. Filipič M, Lӧper P, Niesen B et al. CH3 NH3 PbI3 perovskite/silicon tandem solar cells: characterization based optical simulations. Optics Express 2015, 23, A263--A278.
16. Zhang D, Digdaya IA, Santbergen R. Design and fabrication of a SiOx/ITO double-layer anti-reflective coating for heterojunction silicon solar cells. Sol. Mater. Sol. Cells 2013, 117, 132-138.
17. Green MA, Keevers MJ. Optical properties of intrinsic silicon at 300 K. Prog. Photovolt 1995, 3,189-192.
18. Vogt MR, Hahn H, Holst H et al. Measurement of the optical constants of soda-lime glasses in dependence of iron content and modeling of iron-related power losses in crystalline Si solar cell modules. IEEE J. Photovolt. 2015, 6, 111-118.
19. Vogt MR, Holst H, Schulte-Huxel H et al. Optical constants of UV transparent EVA and the impact on the PV module output power under realistic irradiation. Energy Proc. 2016, 92, 523-530.
20. Escarré J, Sӧderstrӧm K, Despeisse M et al. Geometric light trapping for high efficiency thin film silicon solar cells. Sol. Energy Mater. Sol. Cells 2012, 98, 185-190.
21. Hou F, Han C, Isabella O et al. Inverted pyramidally-textured PDMS antireflective foils for perovskite/silicon tandem solar cells with flat top cell. Sol. Energy Mater. Sol. Cells 2019, 56, 234-240.
22. Wang H, Haroldson R, Balachandran B et al. Nanoimprinted perovskite nanograting photodetector with improved efficiency. ACS nano 2016, 10, 12, 10921—10928.
23. Tockhorn P, Sutt J, Colom R et al. Improved Quantum Efficiency by Advanced Light Management in Nanotextured Solution-Processed Perovskite Solar Cells, ACS Photon. 2020, 7, 9, 2589–2600.
